# Intimate partner violence during the confinement period of the COVID-19 pandemic: exploring the French and Cameroonian public health policies

**DOI:** 10.11604/pamj.supp.2020.35.2.23398

**Published:** 2020-05-28

**Authors:** Joel Noutakdie Tochie, Ingrid Ofakem, Gregory Ayissi, Francky Teddy Endomba, Nkengafac Nyiawung Fobellah, Cyrille Wouatong, Mazou Ngou Temgoua

**Affiliations:** 1 Department of Anesthesiology and Intensive Care, Faculty of Medicine and Biomedical Sciences, University of Yaoundé 1, Yaoundé, Cameroon; 2 Department of Obstetrics and Gynecology, Faculty of Medicine and Biomedical Sciences, University of Yaoundé 1, Yaoundé, Cameroon; 3 Department of Psychiatry, University of Bourgogne, Dijon, France; 4 Department of Internal Medicine and Specialties, Faculty of Medicine and Biomedical Sciences, University of Yaoundé 1, Yaoundé, Cameroon; 5 School of Health and Human Sciences, Higher Institute of the University of Saint Monica, Buea, Cameroon; 6 University of Paris Descartes, Paris, France

**Keywords:** COVID-19, confinement, stay-at-home, intimate partner, violence

## Abstract

Coronavirus disease (COVID-19) is an unprecedented pandemic. COVID-19 is a highly contagious and potentially fatal respiratory infection which has spread within three months of its outbreak to more than 173 countries, causing 3.7 million infections and 256,551 deaths at this writing. Unfortunately, no treatment or vaccine currently exists for COVID-19, although several clinical trials are on-going to find a definite solution to this pandemic. Prevention through public health measures remain the best strategy recommended till date. This prevention involves physical distancing and compulsory confinement at home in several European countries, in the UK and USA. Unfortunately, home confinement decreed in most high-income countries like France has been dangerous for women, victims of psychological, physical and sexual violence from their intimate partner. Violence between intimate partners has become an unintended consequence of the stay-at-home policy against COVID-19. Since the promulgation of a home confinement decreed in many high resource settings (USA, UK, Europe, Canada, Australia, etc), the rate of violence between intimate partners has increased tremendously resulting to the worst scenario, women’s death in some of these countries. The stay-at-home law is not yet a national decree in several low resource settings like Africa, where COVID-19 has not been declared an epidemic in several countries. However, intimate partner violence has been reportedly described as a real violation of women’s right before the advent of the COVID-19 pandemic in the African continent. This commentary highlights the effects of intimate partner violence due to COVID-19 confinement in France and extrapolates what may be the effect of an implementation of a COVID-19 confinement law in Cameroon. Also, the authors suggest recommendations to lessen the burden of intimate partner violence in countries with a stay-at-home policy.

## Commentary

The World Health Organization (WHO) defines domestic violence as any physical, sexual, psychological/emotional assault or threat inflicted on a woman or child/adolescent by an intimate male partner/cohabiting partner, parents, siblings, any family relative or someone well known to the family, or another important person (ex-partner) when such violence often occurs at home. Intimate partner violence (IPV) is a subset of domestic violence. The term IPV is currently used to describe what is called “violence against women”. The US National Institutes of Mental Health Committee on Family Violence has proposed a broader description of IPV as “acts that are physically and emotionally harmful or that carry the potential to cause physical harm and may also include sexual coercion or assaults, physical intimidations, threats to kill or harm, restriction of normal activities or freedom and denial of access to resources” [1]. This latter definition includes the three primary types of IPV: physical, sexual, and emotional/psychological/verbal violence. Intentional use of physical force is encompassed in “physical violence,” and use of force to compel a person to engage in a sexual act is what is referred to as “sexual violence”.

“Emotional”, “psychological or “verbal” violence includes threats, humiliation, control of activities, isolation, offensive name calling and attempts to frighten a woman by her intimate partner. Global estimates of IPV indicate that 30% of ever partnered women worldwide have experienced physical and/or sexual violence by an intimate partner at some point in their lives [2]. Survivors of IPV often sustain benign or life-threatening wounds, fractures, traumatic brain injury and miscariage. Others become illicit drug addicts, alcoholics, or develop severe depressive symptoms and other psychosis warranting care in psychiatric institutions. For women who do not survive from IPV, it has been reported that more than 1.3 million people worldwide die each year as a result of violence in all its forms, accounting for 2.5% of global mortality [3]. A recent epidemic of a highly contagious and potentially fatal respiratory infection called coronavirus 19 disease (COVID-19) emerged in December 2019 in China. The pathogen of COVID-19 is a virus called “severe acute respiratory syndrome-coronavirus 2” (SARS-CoV-2) [4].

Within three months of its outbreak, this life-threatening communicable disease spread around the world so quickly than no unprecedented infection, contaminating 3 992 967 people and claiming 274 985 deaths worldwide as of May 08, 2020. As a result, the WHO declared it a pandemic on March 3, 2020 [4]. This has put all global health institutions such as the CDC and WHO, as well as all ministries of health all over the world on high alert to design public health interventions that can limit the transmission of COVID-19 [4]. Meanwhile, researchers are actively working on its curative and preventive treatment. One of these public health interventions adopted in several European countries, notably France, which has become a COVID-19 epidemic country (176 079 infected people and 26 230 deaths in total registered by May 08, 2020) are the ban on gatherings of more than 100 persons, the temporary closure of academic institutions, non-essential public places and home confinement. Home confinement was decreed by the French government on March 17, 2020.

This law stipulates that French citizens can only leave their home to buy food and medicine, to work for those who cannot work at home and a public walk restricted to once a day for less than an hour on a distance less than a kilometer from their home while wearing a face mask [5]. Consequently, this obviously led to a form of compulsory home “imprisonment” expected to be abolished progressively in France on May 11, 2020 [6]. With more than half of the French population in home confinement due to the COVID-19 pandemic, cases of IPV have increased drastically by 30% [7]. Every three days, a woman is reported to die as a result of IPV in France [8]. Unfortunately, victims are often unable to leave their homes due to financial constraints or because the COVID-19 crisis is hindering their ability to find another shelter to live in. Unlike France, most French-speaking African countries have not reached the level of COVID-19 epidemic countries. For example, at this writing on May 08, 2020, there has been a total of 2267 COVID-19 patients and 108 deaths in Cameroon [9].

Initial measures taken by the Cameroonian government to limit the spread of this infection entailed the temporary closure of academic institutions, the locking of national borders, the suspension of visa services, the mandatory wearing of a face mask, bans on gathering of more than 50 persons and lockdowns of all commercial activities by 6:00 pm. However, restriction on gathering of more than 50 persons and closure of all commercial activities by 6:00 pm were abolished on April 19, 2020 while the other restrictions are still in vigor all over the national territory. The confinement of Cameroonians at home has not been decreed by the government. However, the stay-at-home policy is informally encouraged partly as a means to prevent an epidemic of COVID-19 in Cameroon. With an extensive literature search, to the best of our knowledge there is no report on the effects of the COVID-19 pandemic on IPV in Cameroon. However, unpublished data from Cameroon found a prevalence rate of IPV (not related to COVID-19 confinement) of 40% [10].

Emotional violence was ranked first, sexual violence second and physical third. Identified risk factors for IPV for women were earning or having an intimate partner earning less than 50 000 XAF or 83 dollars or 76 euros monthly [OR= 3.4 (95% = 1.6 - 7.5), P=0.002] and females having an unintended pregnancies [OR=2.7 (95% =1.3-5.6)] [10]. With an already high prevalence of IPV not related to COVID-19 (40%), the implementation of a confinement policy in Cameroon is will likely going to lead to terrific effects on IPV. The lack of a French-Cameroonian epidemiological survey leads to paucity of evidence and hampers a comprehensive appraisal of the magnitude of the burden of IPV. However, the aforementioned arguments are reliable to consider IPV as a major global health concern in countries with stay-at-home policies due to COVID-19. Ministries of social affairs, women empowerment and the police force need to critically look into this situation, especially in France. With regards to Cameroon, government decree of a confinement policy will certainly worsen the already prevailing high IPV and will surely require more careful attention to women´s health and rights if this law is implemented.

The voice of these violated women as a result of the COVID-19 pandemic needs to be heard and some urgent actions need to be taken to avoid one woman dying every three days due to IPV linked to COVID-19 confinement. It is our fervent hope that the progressive uplift of this confinement in France on May 11, 2020 will also go a long way to reduce the amount of physical, emotional/psychological or sexual assault to which these females have been subject to since the beginning of the confinement almost two months ago. Meanwhile, victims of IPV should be encouraged to denounce their partners to the appropriate authorities. Routine screening of IPV, its type and severity using the internationally validated modified NorVold abuse questionnaire (Table 1) by health providers attending to all women and female adolescents in clinical settings for any health condition during this period of COVID-19 confinement may also go a long way for early identification of IPV for appropriate measures to be taken depending on the law in force in each country.

**Table 1:** The modified NorVold abuse questionnaire

	EMOTIONAL ABUSE
Mild abuse	Has your partner/ex-partner systematically and for any longer period trying to repress, degrade or humiliate you? YES/NO
Moderate abuse	Has your partner/ex-partner systematically and by threat or force trying to limit your contacts with others or totally control what you may and may not do, refusing to provide and care for you? YES/NO
Severe abuse	Have you experienced living in fear because your partner systematically and for a longer period has threatened you or somebody close to you? YES/NO
	**PHYSICAL ABUSE**
Mild abuse	Have you experienced your partner/ex-partner hitting you, smacking your face, slapping, pushing or holding you firmly against your will, threatening to abuse you with use of weapon; with no injuries and/or lasting pain? YES/NO
Moderate abuse	Have you experienced your partner/ex-partner hitting you with his fist(s) or with a hard object, kicking you, pushing you violently, giving you a beating, thrashing you, giving you bruises, cuts and/or continuing pain or doing anything similar to you? YES/NO
Severe abuse	Have you experienced your partner/ex-partner threatening your life by, beating up, severe contusions, burns broken bones, head injury, internal injury, permanent injury, trying to strangle you, showing a weapon, use of weapon; wound from weapon or knife or by any other similar act? YES/NO
	**SEXUAL ABUSE**
Mild abuse, no genital contact	Has your partner/ex-partner against your will touched parts of your body other than the genitals in a ‘sexual way’ or forced you to touch other parts of his or her body in a ‘sexual way’? YES/NO
Mild abuse, emotional/ sexual humiliation	Has your partner/ex-partner sexually humiliated you in any other way; e.g. by being forced to watch a porno movie or similar against your will, forced to participate in a porno movie or similar, forced to show your body naked or forced to watch when somebody else showed his/her body naked? YES/NO
Moderate abuse, genital contact	Has your partner/ex-partner against your will touched your genitals, used your body to satisfy him sexually or forced you to touch anybody else’s genitals? YES/NO
Severe abuse, penetration	Has your partner/ex-partner against your will put his penis into your vagina, mouth or rectum or tried any of this; put in or tried to put an object or other part of the body into your vagina, mouth or rectum? YES/NO

## Competing interests

The author declares no competing interests.
